# Intraductal fibroadenoma under the nipple in an 11-year-old female

**DOI:** 10.1186/1746-1596-9-32

**Published:** 2014-02-10

**Authors:** Fumiko Hayano, Sohsuke Yamada, Shigeo Nakano, Teruo Watanabe, Yasuyuki Sasaguri, Sunao Koga

**Affiliations:** 1 Department of Breast Surgery, Fukuoka Wajiro Hospital, Fukuok, Japan; 2 Department of Pathology and Cell Biology, School of Medicine, University of Occupational and Environmental Health, 1-1 Iseigaoka, Yahatanishi-ku, Kitakyushu 807-8555, Japan; 3 Laboratory of Pathology, Fukuoka Wajiro Hospital, Fukuoka, Japan

**Keywords:** Intraductal fibroadenoma, Intracanalicular type fibroadenoma, Phyllodes tumour, Breast

## Abstract

Recently, Chung *et al*. have reported the detailed clinicopathological features of an extremely rare case sharing similar histopathological characteristics with fibroadenomas, phyllodes tumours, intraductal papillomas or ductal adenomas, given the name of intraductal fibroadenomatosis, as an unusual variant of intracanalicular fibroadenoma. Herein we demonstrated a very unusual case of intraductal fibroadenoma of the breast with admixture of components of intracanalicular type fibroadenoma or benign phyllodes tumour and a smaller amount of intraductal papilloma, occupying the one duct and some adjacent ductules, presenting as a well-demarcated nodule.

## Letter to the editor

In 2008, Chung *et al*. have demonstrated the detailed clinicopathological features of an extremely rare case sharing similar histopathological characteristics with fibroadenomas, phyllodes tumors, intraductal papillomas or ductal adenomas, given the name as intraductal fibroadenomatosis, as a very unusual variant of intracanalicular fibroadenoma [1]. Following that, there also have been two interesting reported cases describing pathological features with regard to an intraductal fibroepithelial and papillomatous proliferation corresponding to not only fibroadenomas but phyllodes tumours [2]. Herein we reported an extremely rare case of intraductal fibroadenoma of the breast with admixture of components of intracanalicular type fibroadenoma or phyllodes tumour and a smaller amount of intraductal papilloma, occupying the one duct and some adjacent ductules, presenting as a well-demarcated nodule.

**Figure 1:**
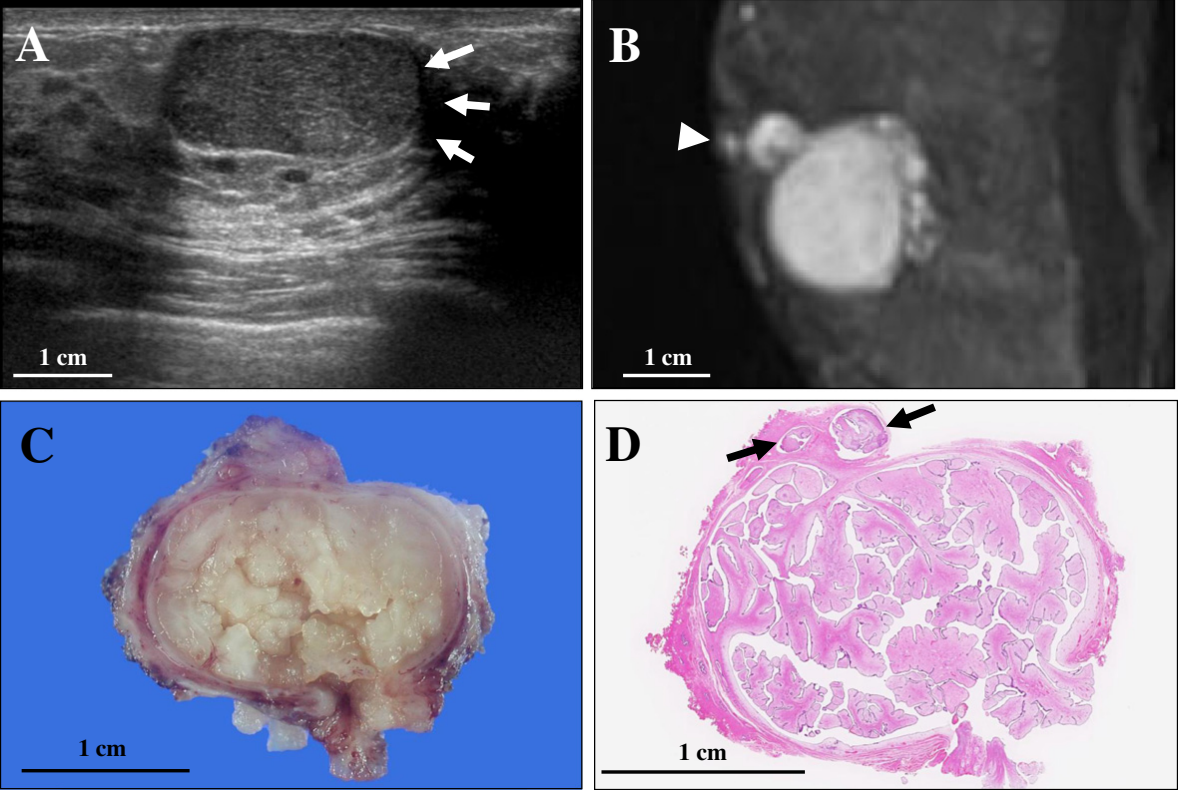
**The findings of ultrasound sonography and MRI scanning at surgery, or gross and microscopic examination of the resected intraductal fibroadenoma specimen. (A)** Ultrasound sonography demonstrated an intracystic well-circumscribed tumour lesion, measuring 22.5 × 21.5 × 11.3 mm in diameter, consisted of a homogeneously solid component with a small amount of surrounding echo-free cystic space (arrows) and overt posterior echo enhancement. Bar = 1 cm. **(B)** MRI showed a mostly homogeneous and hyperintense (white) intracystic well-demarcated nodule partly in a multi-lobulated fashion adjacent to the nipple (arrowhead) on T_2_-weighted images**.** Bar = 1 cm. **(C)** On gross examination, the cut surface revealed a well-circumscribed and encapsulated, peripherally cystic thin cavity-formed, and multi-lobulated soft nodule, measuring 23 × 21 mm in diameter, which looked whitish to yellowish in color and displayed a marked gelatinous appearance. Bar = 1 cm. **(D)** A scanning magnification (H&E stains) showed that the tumor consisted of multiple polypoid papillary lesions in an intracanalicular or leaf-like fashion, partly surrounded by the thinned cystic cavity and displaying the peripheral growth into the terminal ductules (arrowheads). Bar = 1 cm.

**Figure 2:**
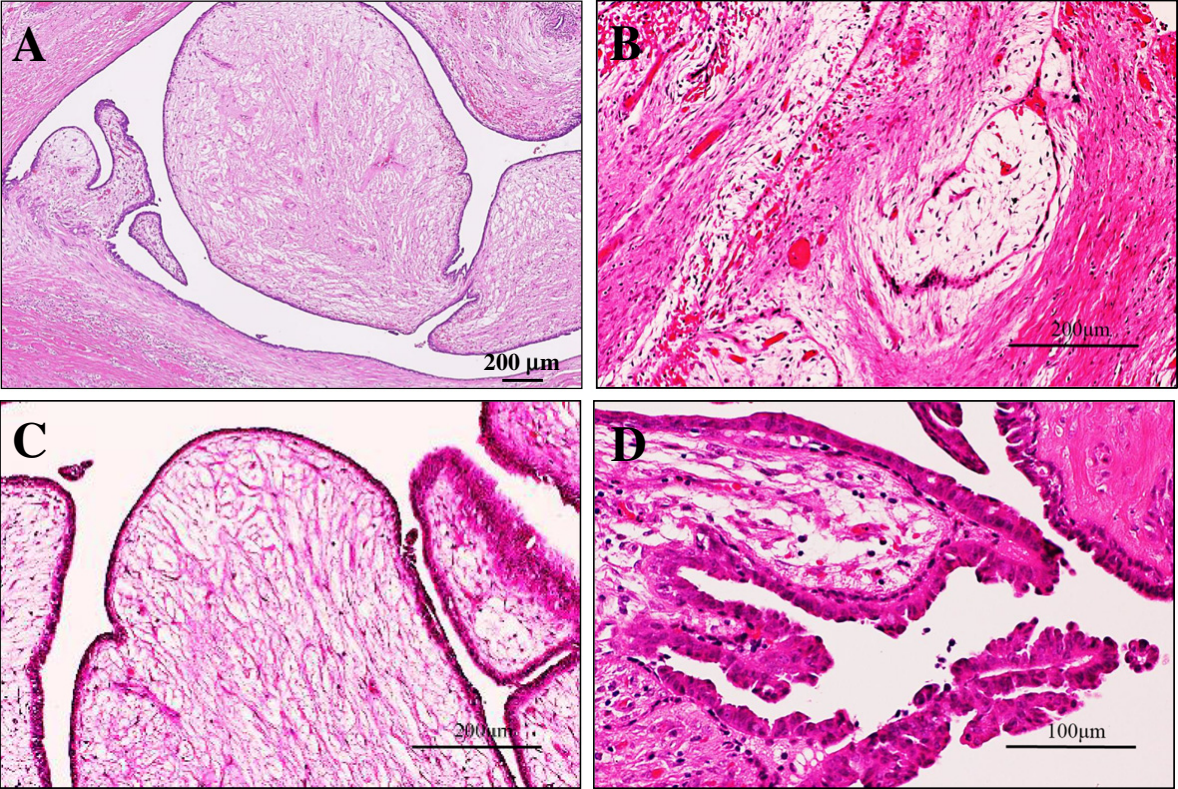
**Microscopic examination of the intraductal fibroadenoma or intradutal phyllodes tumour of the breast. (A)** Low power view showed that its polypoid parts were composed of leaf-like processes with a hypocellular and prominent myxoid stroma, protruding into cystic spaces, reminiscent of intracanalicular type fibroadenoma or benign phyllodes tumour features (H&E stains). Bar = 200 μm. **(B)** In some areas, there were foci of typical intracanalicular variant of fibroadenoma, in which duct lumens were compressed by the proliferating myxoid stroma (H&E stains). Bar = 200 μm. **(C)** On high-power view, those stromal cells showed no significant atypia, but the covering hyperplastic epithelial components were also bland-looking in two cell layers. Mitotic figures were very rarely seen (H&E stains). Bar = 200 μm. **(D)** In others, tiny foci of benign intraductal papilloma with characteristic delicate fibrovascular stalks were rarely seen (H&E stains). Bar = 100 μm.

The patient presented here, an 11-year-old female with an unremarkable previous medical history, had a 2-month history of a gradually enlarging painless and palpable well-circumscribed solitary nodule approximately 2.5 cm in diameter in the nipple of the left breast, accompanied by jelly-like and bloody discharge. Laboratory data, including blood cell count, chemistry and tumor markers, were within normal limits. Ultrasound sonography showed an intracystic well-demarcated tumour lesion, measuring 22.5 × 21.5 × 11.3 mm in diameter, consisted of a homogeneously solid component with a small volume of surrounding echo-free cystic space and overt posterior echo enhancement (Figure 1A). MRI revealed a mostly homogeneous and hyperintense intracystic well-defined nodule partly in a multi-lobulated fashion adjacent to the nipple on T_2_-weighted images (Figure 1B). Moreover, the neck, chest, and abdomen disclosed no definite evidence of tumour lesions including metastatic foci in the lymph nodes or other organs. Surgeons first considered to be a benign intraductal papilloma, however, based on the fine needle biopsy specimens, we interpreted it as a phyllodes tumour, and a simple excision was performed. On gross examination, the cut surface showed a well-circumscribed and encapsulated, peripherally cystic thin cavity-formed, and multi-lobulated soft nodule, measuring approximately 23 × 21 mm in diameter, which looked whitish to yellowish in color and displayed a marked gelatinous appearance (Figure 1C). A scanning magnification demonstrated that the tumour consisted of multiple polypoid papillary lesions in an intracanalicular or leaf-like fashion, partly surrounded by the thinned cystic cavity and displaying the peripheral growth into the terminal ductules (Figure 1D). Excision was diagnosed as complete by this histopathological examination. Microscopically, its polypoid parts were composed of leaf-like processes with a hypocellular and prominent myxoid stroma, protruding into cystic spaces, reminiscent of intracanalicular type fibroadenoma or benign phyllodes tumour features (Figure 2A). In some areas, there were foci of typical intracanalicular variant of fibroadenoma, in which duct lumens were compressed by the proliferating myxoid stroma (Figure 2B). On high-power view, the stromal cells had no significant atypia, but the covering hyperplastic epithelial components were also bland-looking in two cell layers (Figure 2C). Mitotic figures were very rarely seen. In others, tiny foci of benign intraductal papilloma with characteristic delicate fibrovascular stalks were rarely recognized (Figure 2D). By contrast, we could not identify any ductal adenoma elements within our thorough observation [3]. Overall, the main features were of intracanalicular type fibroadenoma or benign phyllodes tumours, admixed with a smaller amount of intraductal papillomas. Immunohistochemically, those epithelial cells were weakly (5% of them) positive for estrogen receptor (ER; BioMed Immunotech, Inc., Tampa, FL, USA, diluted 1:25) and strongly (80% of them) positive for progesterone receptor (PgR; Dako Cytomation Co., Glostrup, Denmark, diluted 1:6), whereas completely negative for HER2 protein (Dako, diluted 1:2). The MIB-1 labeling index (Ki67; Dako, diluted 1:50) was noted in much less than 1% in both of the tumour epithelial and stromal cells, respectively. Based on all these features, we finally made a diagnosis of intraductal fibroadenoma under the nipple, corresponding to the very few similar case reports and letters [1-3]. To date, approximately 6 months routine follow-up after the surgery is established, and the patient remains well and no recurrence has been recognized.

In fact, fibroadenomas or phyllodes tumours are common diseases, compared with some recently published case reports of very rare tumor cell types or very unusual features in the breast [4,5]. Despite that, we report the present extremely rare case, since it is very likely that the current short report of intraductal fibroadenoma located on the subareolar region is clinicopathologically remarkable for two reasons at least. First, our case unusually occurred in very young girl of adolescence, but not adulthood. Actually, Cummings *et al.* have reported that a 13-year-old female case includes foci comprising typical intraductal papillomas, some with leaf-like contours, demonstrating a low-grade phyllodes tumour-like proliferation, admixed with a small amount of fibroadenoma areas [2]. Intraductal fibroadenomas seem to have a special predilection for adolescent female, and might be more common than clinicopathologically considered among the young girls. Second, the present case shares several histopathological characteristics with fibroadenomas, benign phyllodes tumours, and intraductal papillomas, respectively, however, unlike the reported cases [1,2], our main features were of intracanalicular type fibroadenomas or benign phyllodes tumours with conspicuous myxoid stromal change, appearing as leaf-like processes and protruding into intraductal cystic spaces as grossly polypoid lesions. In fact, phyllodes tumours occasionally exhibit intracanalicular growth pattern in elongated and dilated lumina [1,3]. Based on these characteristic clinicopathological features, it is possible that intraductal fibroadenoma might be established as a special new entity of the breast benign tumour. It would be intriguing to study this topic after collecting and investigating a number of cases of intraductal fibroadenoma. The present short case report could interest the scientific community, taken together with some new findings and specific recommendations for histological diagnostics.

## Consent

Written informed consent was obtained from the patient’s parents of kin for the publication of this report and any accompanying images.

## Competing interests

The authors declare that they have no competing interests.

## Authors’ contributions

SY and FH participated in conception of the idea and writing of the manuscript. SY, FH, SN, TW, YS, and SK performed the pathological and immunohistochemical interpretation of the tumor tissue. All authors have read and approved the final manuscript.
